# Identification of potential biomarkers for breast cancer based on salivary metabolomics

**DOI:** 10.3389/fonc.2025.1655213

**Published:** 2025-10-10

**Authors:** Xinyu Jiang, Yumei Jia, Bo Zhang, Kai Yang, Yang Li

**Affiliations:** ^1^ Graduate School, Hunan University of Chinese Medicine, Changsha, China; ^2^ Department of Traditional Chinese Medicine/Integrative Oncology, Hunan Cancer Hospital/The Affiliated Cancer Hospital of Xiangya School of Medicine Central South University, Changsha, China

**Keywords:** breast cancer, caffeine metabolism, salivary metabolomics, biomarkers, cancer

## Abstract

**Introduction:**

Breast cancer (BC) remains the most prevalent malignant tumor in women worldwide and a leading cause of cancer-related mortality. Early screening is essential to improve prognosis, yet current diagnostic methods are often invasive or lack sensitivity. Saliva is an accessible and non-invasive biofluid containing various metabolites that reflect systemic physiological and pathological changes. Thus, salivary metabolomics may provide novel insights into breast cancer-associated metabolic alterations and support the development of early diagnostic strategies.

**Objectives:**

To explore the salivary metabolomic profile of breast cancer patients and identify potential non-invasive biomarkers for early breast cancer screening.

**Methods:**

Saliva samples were collected from a screening set consisting of 30 BC patients and 20 normal controls (NC) volunteers. untargeted metabolomics approach was performed using liquid chromatography–tandem mass spectrometry (LC-MS/MS). Principal component analysis (PCA) and orthogonal partial least squares-discriminant analysis (OPLS-DA), along with KEGG pathway enrichment and receiver operating characteristic (ROC) curve analyses, were employed to characterize metabolic differences and identify potential biomarkers. Additionally, saliva samples from a validation set (52 BC patients and 52 NC volunteers) were collected. Enzyme-linked immunosorbent assay (ELISA) was used to quantify potential biomarkers, and their diagnostic performance was evaluated through ROC curve analysis.

**Results:**

A total of 101 differential metabolites were identified, including 81 upregulated and 20 downregulated compounds. Screening identified 2-aminonicotinic acid and theobromine as potential diagnostic biomarkers. Analysis of the validation set demonstrated that 2-aminonicotinic acid (AUC: 0.81, cut-off: 5.88 ng/mL) and theobromine (AUC: 0.75, cut-off: 5.27 ng/mL) exhibit promising diagnostic potential.

**Conclusion:**

The salivary metabolome of breast cancer patients displays distinct changes compared to healthy individuals. Salivary 2-aminonicotinic acid and theobromine emerge as promising non-invasive biomarkers for breast cancer detection. Nevertheless, larger-scale validation studies are warranted to substantiate their specificity and clinical utility.

## 1 Introduction

Breast cancer (BC) is the most common malignant tumor among women, ranking first in both incidence and mortality among female malignancies. According to a report by the World Health Organization (WHO), more than 2.3 million new breast cancer cases were diagnosed globally in 2020, with approximately 685,000 related deaths (1). These figures not only underscore the severe threat breast cancer poses to women’s health worldwide but also highlight its significance and urgency in public health (2). The high incidence and mortality rates of breast cancer make it one of the major challenges to women’s health (3). There is an urgent need to enhance research and application of early screening, diagnosis, and treatment strategies. Currently, breast cancer detection primarily relies on imaging techniques such as mammography, ultrasound, and magnetic resonance imaging (MRI) (4), as well as invasive procedures like tissue biopsy or minimally invasive blood tests (5). Although these technologies play a crucial role in diagnosis, they often fail to fully meet the need for non-invasive, convenient, and efficient screening, particularly for the early detection of breast cancer (6). Therefore, developing a novel non-invasive diagnostic method is urgently needed.

Metabolomics, as a powerful analytical tool, has been widely used to study the expression changes of metabolites in complex human diseases. Its high specificity and sensitivity offer distinct advantages in research across various diseases (7). By quantitatively analyzing the overall metabolic profile changes of organisms under normal physiological conditions, pathological processes, or external stimuli, metabolomics provides an indispensable platform for discovering potential biomarkers (8). This technology not only reveals disease-related metabolic pathway abnormalities but also provides critical scientific evidence for early diagnosis, prognosis assessment, and the development of personalized treatment strategies. In recent years, the application of metabolomics in cancer research has expanded significantly. Untargeted metabolomics and lipidomics studies using liquid chromatography-tandem mass spectrometry (LC-MS/MS) have shown tremendous potential in discovering novel biomarkers and uncovering metabolic changes (9). Mohit Jain et al. used LC-MS/MS technology to analyze the consumption and release (CORE) curves of 219 metabolites in the culture media of the NCI-60 cancer cell lines, including breast cancer cells, revealing unique metabolic characteristics of cancer cells (10). In further studies, they employed RRLC-MS/MS to successfully differentiate between BC patients and normal control (NC volunteers), identifying 12 potential breast cancer biomarkers in urine samples (11). It is worth noting that, in addition to urinary metabolomics, salivary metabolomics has gained increasing attention in recent years and has shown promising diagnostic potential. Sugimoto et al. used CE-MS technology to analyze saliva samples and identified 14 amino acids as potential biomarkers for breast cancer diagnosis (12). Moreover, researchers have used salivary biomarkers to diagnose diseases such as oral cancer (13), pancreatic cancer (14), and lung cancer (15). However, despite the growing body of research on salivary metabolomics in other cancer types, its application in breast cancer remains relatively limited, with the associated metabolic characteristics not yet systematically analyzed. Further in-depth research is urgently needed to clarify its clinical application value.

Saliva, as a bodily fluid rich in various metabolites, offers distinct advantages in disease screening and early diagnosis due to its non-invasive collection, ease of use, and high reproducibility (16). Existing studies have shown significant metabolic abnormalities in breast cancer patients, including disruptions in glucose metabolism, amino acid metabolism, and lipid metabolism (17). These metabolic changes may be reflected in saliva through the bloodstream or other pathways. Therefore, by integrating salivary metabolomics, it is possible to identify new non-invasive biomarkers for the early diagnosis of breast cancer, providing a more convenient screening method for clinical use.

This study, by combining the non-invasive approach of salivary metabolomics, aims to develop a more accurate, non-invasive, and easily scalable breast cancer detection method. This approach not only has the potential to improve early diagnosis rates but also reduce patient suffering and healthcare costs, offering a new breakthrough in BC prevention and control. Furthermore, emerging evidence has suggested that salivary metabolomics may reflect systemic metabolic changes associated with tumor progression through immune and endocrine signaling pathways (12), thereby supporting its potential use in early cancer detection. This theoretical foundation provides a rationale for exploring salivary biomarkers as viable tools for breast cancer screening, although further empirical studies are required to establish their specificity and clinical utility (18).

## 2 Materials and methods

### 2.1 Clinical samples and ethical approval

All participants were recruited from the Department of Integrative Oncology at Hunan Provincial Cancer Hospital between December 2024 and August 2025, where saliva samples were collected for subsequent analytical evaluation. Subjects were enrolled based on stringent inclusion and exclusion criteria. Breast cancer diagnoses were confirmed through established clinical and histopathological standards. The control group comprised age- and ethnicity-matched (all Han Chinese) women with no history of malignancy or breast-related disorders. Inclusion criteria for breast cancer patients encompassed histologically confirmed breast cancer, absence of prior systemic therapy at the time of saliva collection, and no concurrent malignancies. Exclusion criteria for controls included a history of cancer, autoimmune disorders, or recent infections. The screening set consisted of 30 BC patients and 20 NC volunteers (**Table 1**). The sample size for the validation set was determined using MedCalc software based on area under the curve (AUC) results from the screening set (α = 0.05, β = 0.2, null hypothesis value = 0.7), yielding 52 BC patients and 52 NC volunteers (**Supplementary Table S1**). All participants provided written informed consent. This study was approved by the Ethics Committee of Hunan Provincial Cancer Hospital (Approval No. 2025-KY-KS-045) and conducted in accordance with the Declaration of Helsinki.

**Table 1:** Clinic characteristics of the screening set.

Characteristic	BC Patients (n=30)	Controls (n = 20)	p-value
Ages	41 ± 10.5	40 ± 5.1	0.9
BMI (kg/mue	28.5 ± 3.2	27.4 ± 2.3	0.88
Ethnicity	Chinese	Chinese	
Gender (Male/Female)	0/30	0/20	
Menopausal Status, n (%)			0.594
- Premenopausal	3 (10%)	3 (15%)	
- Postmenopausal	27 (90%)	17 (85%)	
Clinical stage			N/A
Early stage (I-II)	20 (I:6, II:14)	N/A	
Advanced stage (III-IV)	10 (III:8, IV:2)	N/A	
TNM status			N/A
Tumor status (T)	T1:13, T2:16, T3:0, T4:1	N/A	
Regional lymph node status (N)	N0:16, N1:6, N2:6, N3:2	N/A	
Distant metastasis status (M)	M0:29, M1:1	N/A	

p-value derived from t-test for continuous variables and Chi-square test for categorical variables comparing BC patients vs. Healthy Controls. N/A, Not Applicable.

### 2.2 Reagents and instruments

The reagents used in this study are listed in **Table 2**. The instruments used in this study are summarized in **Table 3**.

**Table 2:** Reagents.

Name	CAS Number	Purity	Brand
Methanol	67-56-1	LC-MS grade	CNW Technologies
Acetonitrile	75-05-8	LC-MS grade	CNW Technologies
Ammonium acetate	631-61-8	LC-MS grade	SIGMA-ALDRICH
Ammonium hydroxide	1336-21-6	LC-MS grade	CNW Technologies
Ultrapure water (ddH2O)	–	–	Watsons
Acetic acid	64-19-7	LC-MS grade	SIGMA-ALDRICH
2-Propanol	67-63-0	LC-MS grade	

**Table 3:** Instruments.

Instrument	Model	Brand
Ultra-high performance liquid chromatography (UHPLC)	Vanquish	Thermo Fisher Scientific
High-resolution mass spectrometer (HRMS)	Orbitrap Exploris 120	Thermo Fisher Scientific
Centrifuge	Heraeus Fresco17	Thermo Fisher Scientific
Analytical balance	BSA124S-CW	Sartorius
Ultrasonic cleaner	PS-60AL	Shenzhen Redbang Electronics Co., Ltd.
Homogenizer	JXFSTPRP-24	Shanghai Jingxin Technology Co., Ltd.
Microplate reader	SpectraMax M5	Molecular Devices, LLC
Freeze dryer	LGJ-10C	Sihuan Frey Technology Development Co., Ltd.

### 2.3 Saliva collection and processing

Participants were instructed to abstain from consuming coffee, chocolate, cakes, and other refined sweets for one week prior to sample collection. Additionally, they were advised to avoid eating, drinking, smoking, or using oral hygiene products for at least one hour before the collection of samples. They rinsed their mouths thoroughly with deionized water and expelled any residual saliva. Participants were seated comfortably with their eyes open, head slightly tilted forward, and instructed to rest for 5 minutes to minimize facial movements. Saliva was collected for 5 minutes using expectoration: participants were asked to accumulate saliva at the bottom of their mouths and expel it into a 50 mL centrifuge tube every 60 seconds (with a reminder not to expectorate mucus). The saliva samples were then centrifuged at 4°C, 2600 g for 15 minutes. The supernatant was quenched in liquid nitrogen and stored at -80°C.

### 2.4 Experimental methods

#### 2.4.1 Metabolite extraction

Samples were thawed on ice and subjected to metabolite extraction using the Starlid™ automated workstation. A 100 µL aliquot of each sample and 400 µL of extraction solvent (methanol: acetonitrile = 1:1, v/v, containing isotopically labeled internal standards) were transferred to a 96-well protein precipitation plate. The mixture was vortexed at 750 rpm for 5 minutes, left to stand for 5 minutes, filtered, and the filtrate was collected. An equal volume of supernatant from all samples was mixed to create a quality control (QC) sample for analysis.

#### 2.4.2 Instrumental analysis

For polar metabolites, an ultra-high-performance liquid chromatography (UHPLC) system, Vanquish (Thermo Fisher Scientific), was used in conjunction with a Waters ACQUITY UPLC BEH Amide (2.1 mm × 50 mm, 1.7 μm) column for chromatographic separation of target compounds. The mobile phase consisted of A: water with 25 mmol/L ammonium acetate and 25 mmol/L ammonia, and B: acetonitrile. The sample tray was maintained at 4°C, and the injection volume was 2 µL. The Orbitrap Exploris 120 mass spectrometer, controlled by Xcalibur software (version 4.4, Thermo), was used for data acquisition in both full MS and MS/MS modes. Detailed parameters are as follows: Sheath gas flow rate: 50 Arb; Aux gas flow rate: 15 Arb; Capillary temperature: 320°C; Full MS resolution: 60,000; MS/MS resolution: 15,000; Collision energy: SNCE 20/30/40; Spray voltage: 3.8 kV (positive) or -3.4 kV (negative).

### 2.5 Detection of 2- aminonicotinic acid and theobromine by ELISA

2-aminonicotinic acid and theobromine ELISA kits were purchased by from Cloud-Clone Corp. (Wuhan, China). To detect 2-aminonicotinic acid and theobromine in saliva using ELISA, thaw samples stored at -80°C on ice and centrifuge at 2600g for 5 minutes at 4°C. Dilute supernatant 1:5 in PBS to reduce matrix effects. Add 100 µL of diluted samples and standards to a 96-well ELISA plate pre-coated with specific antibodies, then incubate for 1–2 hours at room temperature. Wash plates, add biotinylated detection antibodies, followed by streptavidin-HRP conjugate, and develop with TMB substrate. Measure absorbance at 450 nm using a microplate reader.

### 2.6 Data analysis

Principal component analysis (PCA) and orthogonal partial least squares discriminant analysis (OPLS-DA) were performed using R-4.1.1. The importance of variables in the OPLS-DA model was assessed using the variable importance in projection (VIP) scores, and differential metabolites were selected based on VIP > 1, p < 0.05, and fold change (FC). The metabolites were annotated using the KEGG compound database and mapped to KEGG pathways for pathway analysis. Finally, the diagnostic potential of significantly different metabolites was evaluated using ROC curves. The software and analysis tools used in this study are summarized in **Table 4**.

**Table 4:** Data analysis software.

Analysis	Software (Version)
PCA	SIMCA (18.0.1)
OPLS-DA	SIMCA (18.0.1)
OPLS-DA Permutation Test	R (ggplot2) (3.3.5)
Volcano Plot	R (ggplot2) (3.3.5)
Z-score Plot	R (ggplot2) (3.3.5)
Stick Plot	R (ggplot2) (3.3.5)
Correlation Heatmap	R (corrplot) (0.89)
Chord Diagram	R (ggraph) (2.0.5)
KEGG Annotation Plot	R (base) (3.6.3)
KEGG Pathway Annotation Classification	R (ggplot2) (3.3.5)
KEGG Enrichment Plot	R (ggplot2) (3.3.5)
Metabolic Pathway Bubble Plot	R (KEGGgraph, ggplot2) (1.46.0, 3.3.5)
Metabolic Pathway Tree Diagram	R (KEGGgraph, treemap) (1.46.0, 2.4-2)
ROC Curve Plot	R (plotROC, pROC) (2.2.1, 1.16.2)

## 3 Results

### 3.1 Principal component analysis

**Figure 1:**
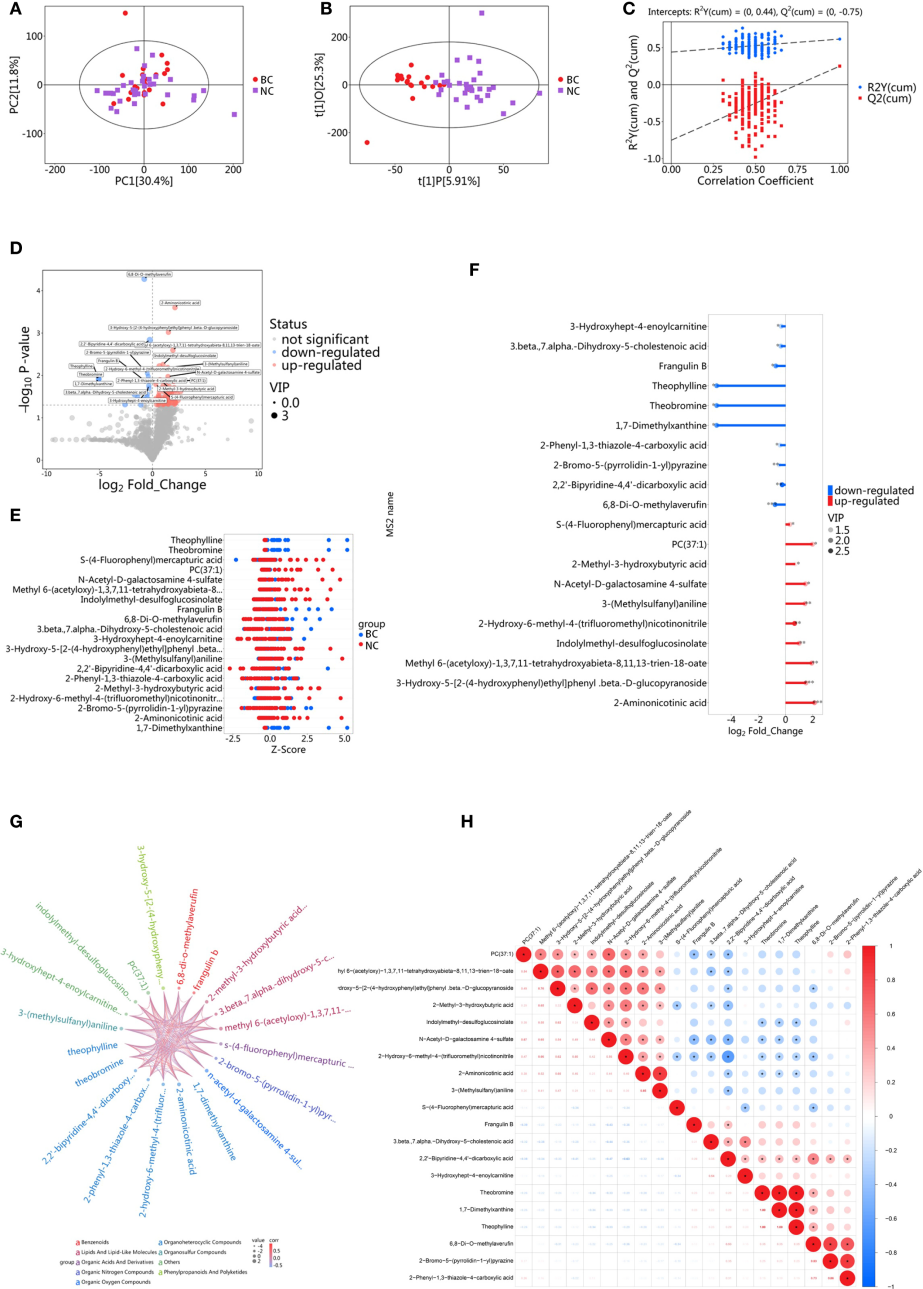
Metabolomic analysis of saliva in the screening set. **(A)** PCA of BC group and NC group; **(B)** OPLS-DA analysis of BC group and NC group; **(C)** Results of permutation test for OPLS-DA model; Ry(cum) represents the cumulative explained variance in the y-direction of the model; Q(cum) represents the proportion of the predicted variance of the model; **(D)** Volcano plot for differential metabolite screening; **(E)** Z-score plot for differential metabolites; **(F)** Stick diagram of differential metabolites; **(G)** Chord diagram for correlation analysis of differential metabolites; **(H)** Heatmap for correlation analysis of differential metabolites.

The PCA score plot showed that the screening set samples were primarily distributed within the 95% confidence interval, and the QC samples exhibited good clustering, indicating the high stability and reliability of the experimental data (**Supplementary Figure S1**). PCA of screening set samples from the BC group and the NC group is presented in **Figure 1A**. To further distinguish the metabolic differences between BC patients and NC volunteers, OPLS-DA was performed. The OPLS-DA score plot (**Figure 1B**) revealed that the model’s R²X, R²Y, and Q values were 0.312, 0.617, and 0.251, respectively, with clear separability between the two groups. The most significant metabolic features contributing to the group differences were identified. To validate the model’s reliability, a permutation test (n=200) was conducted, where the class variable Y’s arrangement was randomly shuffled to generate random models with R² and Q² values (**Figure 1C**). The results indicated that the model was partial overfitted and showed high statistical significance. Based on the screening criteria (VIP > 1 and P < 0.05), 101 differential metabolites were selected from the preliminary analysis of metabolic features, of which 81 were significantly upregulated and 20 were significantly downregulated (**Figure 1D**). To further analyze the distribution of these differential metabolites across groups, a Z-score analysis was performed on the top 10 most significantly upregulated and downregulated metabolites (**Figure 1E**). The results revealed a clear difference in the distribution of metabolites between the two groups. Notably, the most significantly upregulated metabolites included 2-Aminonicotinic acid, N-Acetyl-D-galactosamine 4-sulfate, and 6,8-Di-O-methylaverufin, while the most significantly downregulated metabolites were Theophylline, 1,7-Dimethylxanthine, and 3-Hydroxyhept-4-enoylcarnitine. A stick chart further confirmed that 2-Aminonicotinic acid (upregulated) and Theophylline (downregulated) were the most significantly different metabolites between the two groups (**Figure 1F**). Correlation analysis of the differential metabolites revealed a strong positive correlation between 2-Methyl-3-hydroxybutyric acid and 2-Methylbutyric acid, with a correlation coefficient close to 1 (**Figures 1G, H**), suggesting that these two metabolites may act synergistically in the same biological process or be regulated by similar mechanisms. On the other hand, the correlation coefficient between Theophylline and Caffeine metabolism was negative and of substantial magnitude, indicating an inverse trend in their changes under experimental conditions. This may reflect their roles in different biological pathways or opposing regulatory influences under the experimental conditions.

### 3.2 Pathway analysis

**Figure 2:**
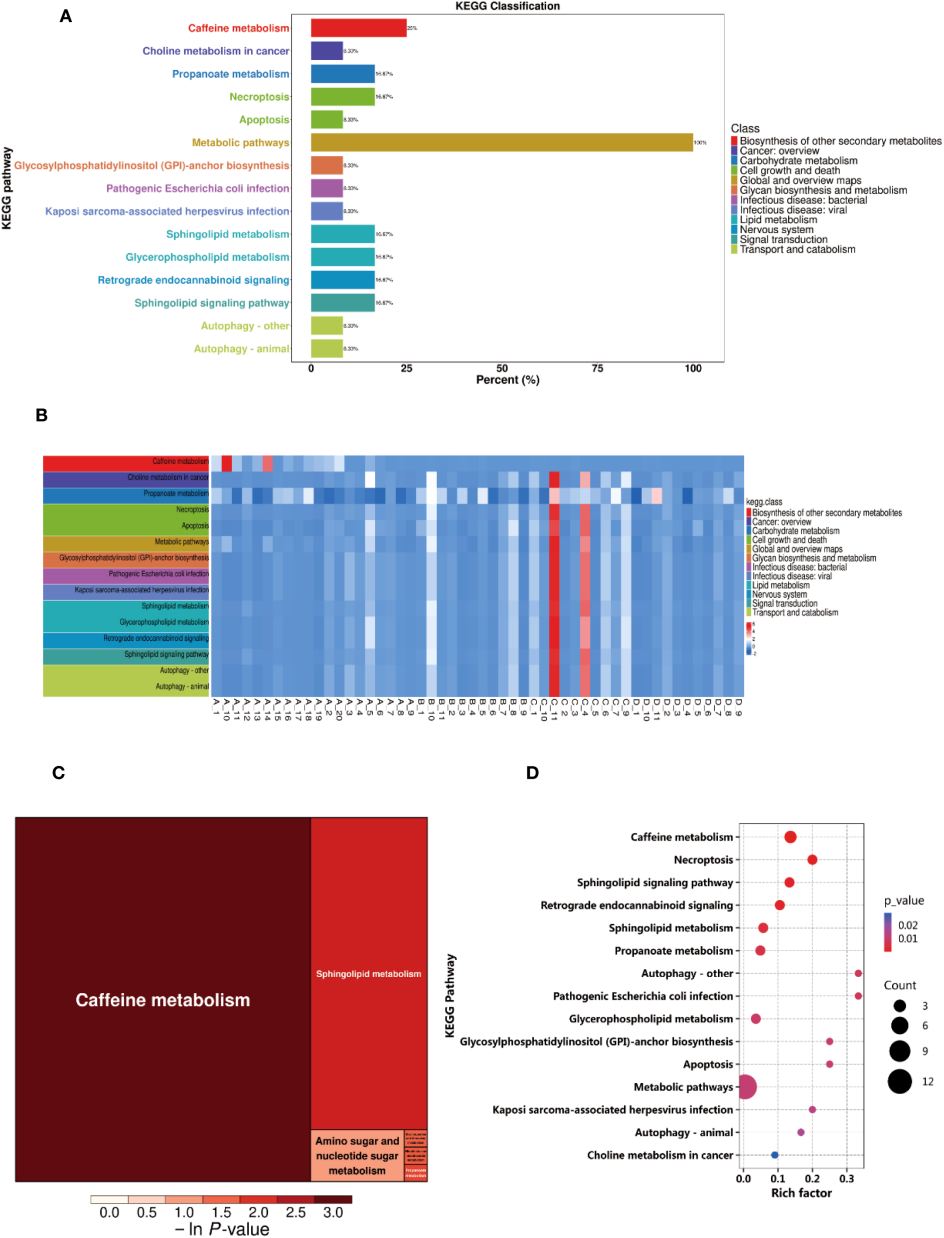
Differential metabolite pathway analysis. **(A)** KEGG Enrichment Analysis Plot. **(B)** KEGG Heatmap. **(C)** Rectangular Tree Map of Differential Metabolite Pathways. **(D)** Bubble Plot of Differential Metabolite Pathways.

To explore the metabolic changes between BC patients and NC volunteers and their biological significance, this study performed pathway annotation and enrichment analysis of the differential metabolites based on the KEGG database. The KEGG enrichment classification plot (**Figure 2A**) revealed that the differential metabolites were primarily enriched in pathways such as the biosynthesis of other secondary metabolites, cancer overview, and carbohydrate metabolism. These findings suggest that these pathways may play a significant role in the metabolic reprogramming of breast cancer. Additionally, the KEGG heatmap (**Figure 2B**) displayed the expression level changes of metabolites across the pathways, with color gradients from blue to red indicating increasing metabolite abundance. This further highlighted the metabolic differences between BC patients and healthy controls. Further KEGG pathway enrichment analysis indicated significant differences in several metabolic pathways between the experimental groups, with the highest proportions observed in caffeine metabolism, sphingolipid metabolism, and propanoate metabolism (**Figure 2C**). The KEGG enrichment bubble plot (**Figure 2D**) visually depicted the enrichment of these pathways, with caffeine metabolism and choline metabolism showing higher Rich Factors and significant P-values (*P* < 0.05), suggesting that these pathways may be significantly activated in BC.

### 3.3 ROC analysis of differential metabolites

**Figure 3:**
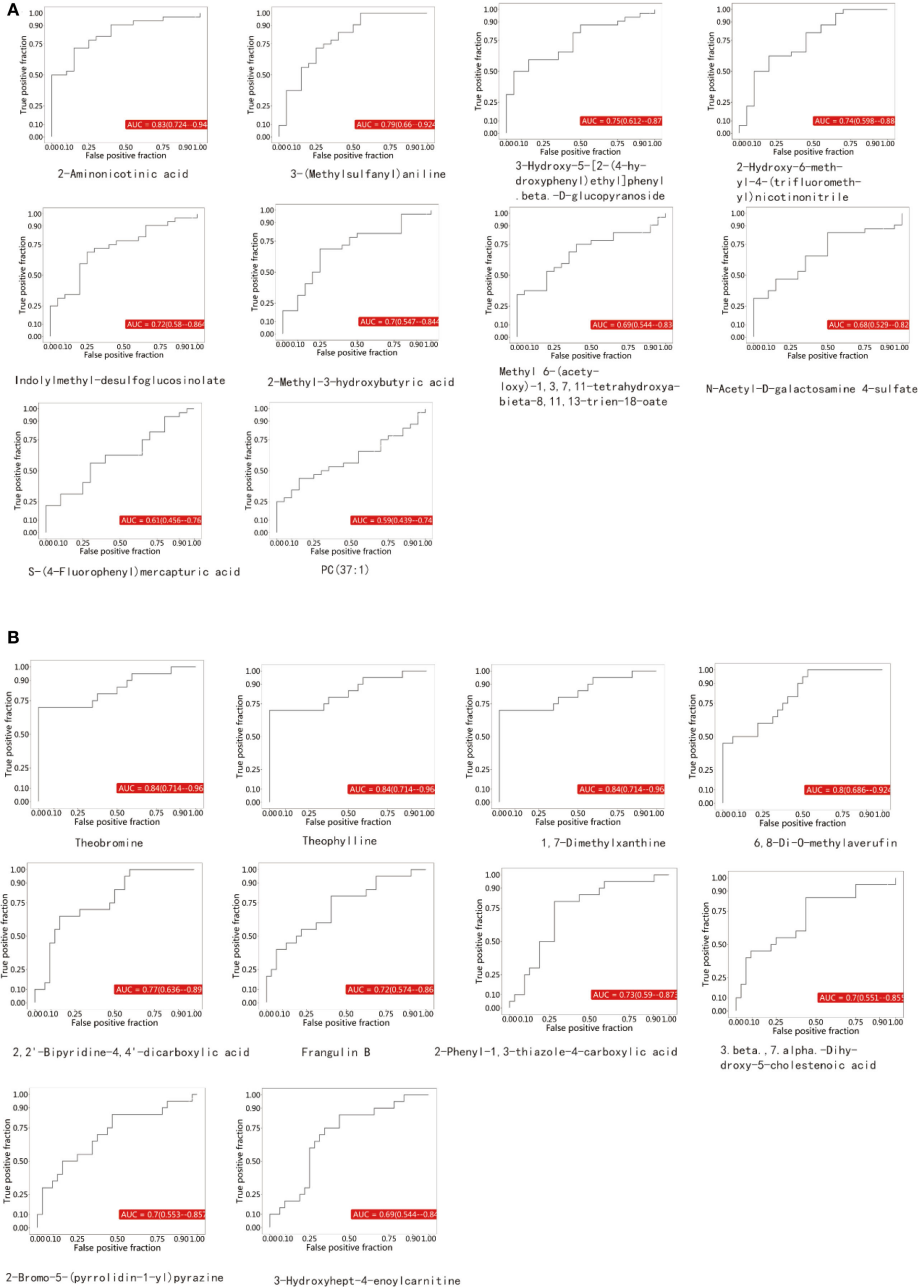
**(A)** represents the upregulated metabolites, while **(B)** represents the downregulated metabolites.

ROC curves were constructed for each comparison based on a series of binary classifications (defined by threshold values), with the true positive rate (sensitivity) plotted on the vertical axis and the false positive rate (1-specificity) on the horizontal axis. For each clearly identified differential metabolite, we plotted its ROC curve and calculated the AUC. The AUC value ranges between 0.5 and 1.0. An AUC closer to 1 indicates better diagnostic performance. An AUC between 0.5 and 0.7 reflects low accuracy, between 0.7 and 0.9 indicates moderate accuracy, and above 0.9 suggests high accuracy. Among the upregulated metabolites in this study, 2-Aminonicotinic acid exhibited the highest AUC value (**Figure 3A**), suggesting its potential as a biomarker for breast cancer. Furthermore, among the downregulated metabolites, Theobromine showed the highest AUC value (**Figure 3B**), indicating its potential diagnostic value in distinguishing between BC group and NC group in the screening set.

### 3.4 Validation of caffeine and 2-aminonicotinic acid as potential biomarkers

**Figure 4:**
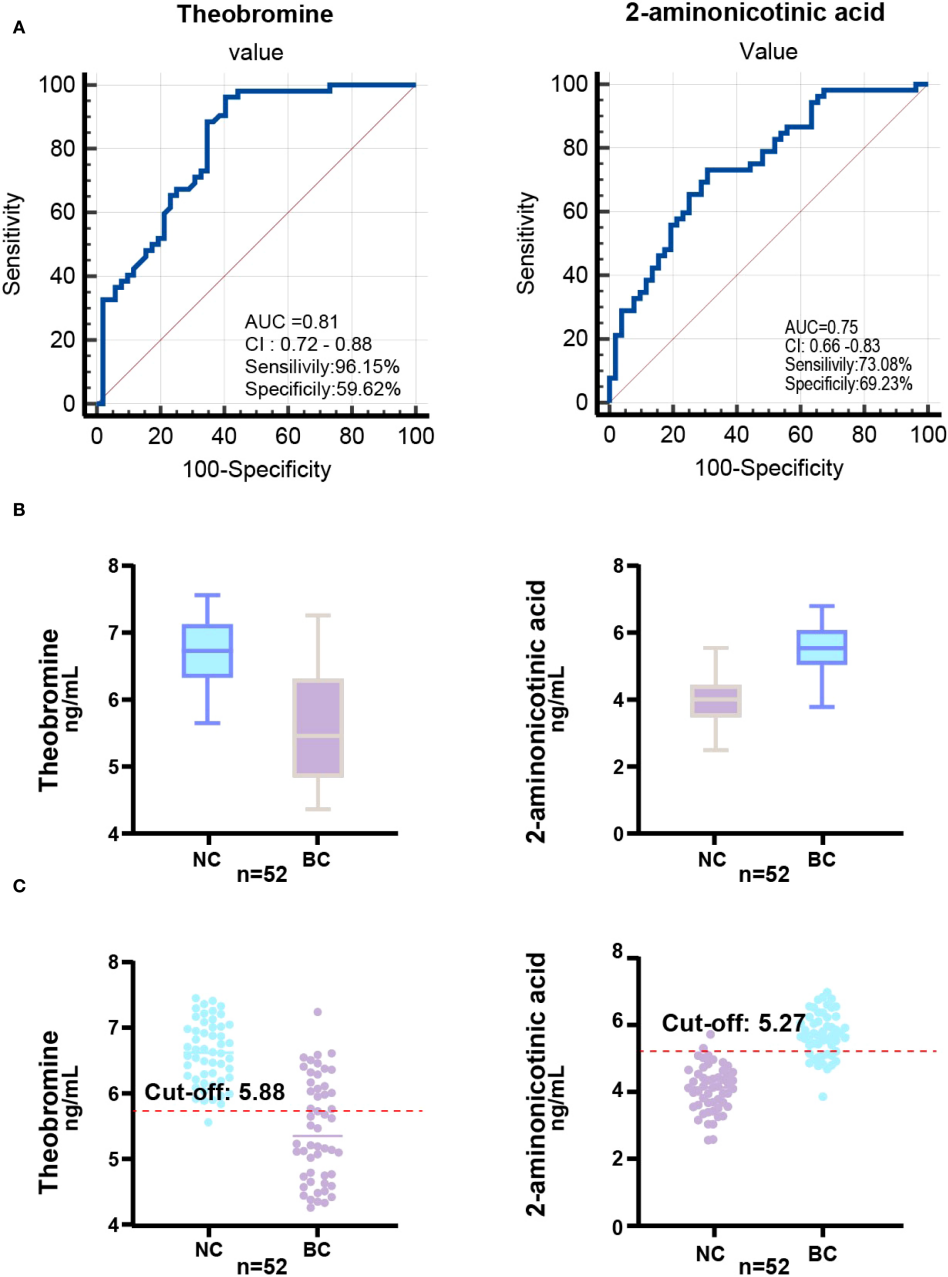
ROC analysis and biomarker distribution in the validation set. **(A)** ROC curve illustrating the AUC, sensitivity, and specificity of the Theobromine and 2-Aminonicotinic acid in the validation set (52 NC *vs.* 52 BC) based on saliva concentrations. **(B)** Box-plot showing Theobromine and 2-Aminonicotinic acid levels in validation set. **(C)** Scatter plot showing the relationship between Theobromine and 2-Aminonicotinic acid levels in the validation set, with the optimal cut-off value determined for diagnostic classification.

To validate the diagnostic potential of theobromine and 2-aminonicotinic acid, identified as significantly downregulated and upregulated metabolites, respectively, in the screening set through OPLS-DA analysis, we further quantified their concentrations in a larger cohort comprising 52 BC patients and 52 NC volunteers. The levels of theobromine and 2-aminonicotinic acid in saliva samples were measured using ELISA. ROC analysis was performed to evaluate the discriminative capacity of each metabolite and to determine the optimal cutoff value for maximizing sensitivity and specificity (**Figure 4A**). For theobromine, ROC analysis yielded an AUC of 0.81 (95% CI: 0.72–0.88), indicating robust discriminative ability between BC patients and NC volunteers. The optimal cutoff value for theobromine concentration was 5.88 ng/mL, with a sensitivity of 96.15% and a specificity of 59.62%. For 2-aminonicotinic acid, ROC analysis revealed an AUC of 0.75 (95% CI: 0.66–0.83), suggesting excellent discriminative performance. The optimal cutoff value was 5.27 ng/mL, with a sensitivity of 73.08% and a specificity of 69.23%.

Box-plots of theobromine and 2-aminonicotinic acid concentrations (**Figure 4B**) demonstrate a significant reduction in theobromine levels in BC group compared to NC group. Conversely, 2-aminonicotinic acid levels were significantly elevated in BC group relative to controls. Scatter plots of individual sample concentrations (**Figure 4C**) further confirm the distinct separation between the two groups. The optimal cutoff values for theobromine and 2-aminonicotinic acid effectively discriminated between the BC group and NC group. These findings validate theobromine and 2-aminonicotinic acid as reliable salivary biomarkers for distinguishing BC patients from healthy controls, supporting their potential utility in non-invasive breast cancer screening.

## 4 Discussion

This study employed LC-MS/MS non-targeted metabolomics to deeply analyze the salivary metabolic profiles of BC patients and NC volunteers, successfully identifying a range of potential biomarkers with diagnostic value. We systematically identified differential metabolites using PCA, OPLS-DA, and permutation testing. The OPLS-DA model demonstrated potential overfitting. Given the complexity of biological data in metabolomics studies, these differential metabolites warrant further investigation. To mitigate the risk of overfitting, stringent filtering criteria (VIP > 1 and *p* < 0.05) were applied. Theobromine and 2-aminonicotinic acid were identified as significantly upregulated or downregulated in BC patients, suggesting their potential roles in BC onset and progression. Additionally, ROC analysis further validated the diagnostic potential of theobromine and 2-aminonicotinic acid. In the validation cohort, high AUC values for theobromine and 2-aminonicotinic acid indicated their ability to effectively discriminate BC patients from healthy individuals, exhibiting potential diagnostic sensitivity and specificity. Theobromine, a key intermediate in the caffeine metabolism pathway, was minimally influenced by dietary factors due to pre-collection dietary restrictions. Evidence suggests that alterations in theobromine levels may be closely associated with changes in cytochrome P450 enzymes (e.g., CYP1A2) in BC patients.

This suggests that the altered caffeine metabolism pathway may contribute to the metabolic reprogramming observed in breast cancer. Additionally, 2-Aminonicotinic acid, a derivative of nicotinic acid, could be implicated in the altered nicotinamide metabolism observed in cancer cells. Given that these metabolic pathways are known to be involved in energy metabolism, oxidative stress, and cell signaling, these findings point to the significant role of these metabolites in cancer pathophysiology. Studies have shown that the inhibition of CYP1A2 activity has been reported in various cancers, which may result in abnormal accumulation or excessive consumption of caffeine and its metabolites, such as theobromine (19). Additionally, dysregulation of theobromine metabolism may reflect an increase in oxidative stress levels within the tumor microenvironment, which plays a crucial role in the metabolic adaptation and survival strategies of breast cancer cells (20). Additionally, 2-Aminonicotinic acid, a key product of nicotinic acid metabolism, may serve as an indicator of abnormal nicotinic acid metabolic pathways in breast cancer patients (21). Nicotinic acid and its derivatives play vital roles in cellular energy metabolism and DNA repair, and their metabolic imbalance may impact the proliferation and survival of tumor cells (22, 23). Notably, the abnormal levels of 2-Aminonicotinic acid may be associated with dysregulation of nicotinamide adenine dinucleotide (NAD^+^) metabolism, which is widely recognized as one of the key mechanisms in cancer metabolic reprogramming (24). These metabolic alterations may be driven by systemic immune and endocrine signaling pathway changes induced by the tumor, as previously reported in salivary metabolomics studies (14). The interplay between purine and nicotinic acid metabolism underscores their potential as diagnostic biomarkers, warranting further investigation to elucidate their mechanistic roles in breast cancer progression (25).

In pathway analysis, significant differences were observed between the salivary metabolomes of breast cancer patients and healthy individuals, particularly in key metabolic pathways such as caffeine metabolism, sphingolipid metabolism, and propanoate metabolism. This study found that the caffeine metabolism, sphingolipid metabolism, and propanoate metabolism pathways contributed the most to the metabolic differences, which aligns with the phenomenon of metabolic reprogramming in breast cancer. The caffeine metabolism pathway also showed significant changes in breast cancer patients. Caffeine is primarily metabolized by cytochrome P450 enzymes, particularly CYP1A2, whose activity is suppressed in various cancers. Caffeine metabolism is not only closely linked to energy metabolism but also plays a role in other biological processes (26). Additionally, caffeine metabolism is not only closely related to energy metabolism, but its metabolites may also be linked to increased oxidative stress levels in the tumor microenvironment. Previous studies have shown that breast cancer cells can promote their survival and proliferation by regulating their redox state, and further influence cancer cell proliferation and drug resistance through the modulation of cell signaling pathways (27). Specifically, caffeine and its metabolites can inhibit cancer cell proliferation and induce apoptosis by suppressing the PI3K/AKT/mTOR signaling pathway (28). Additionally, caffeine metabolism regulates the AMPK signaling pathway, affecting energy metabolism and autophagy in cancer cells (29).

In this study, the significant changes in caffeine metabolism suggest that breast cancer cells may adapt to their energy demands and drug resistance by regulating caffeine metabolism. This finding provides new insights into the metabolic regulatory mechanisms of breast cancer and indicates that the caffeine metabolism pathway could serve as a potential therapeutic target. The importance of changes in sphingolipid metabolism in breast cancer has been widely recognized. Sphingolipid molecules, such as sphingosine-1-phosphate (S1P), play a key role in regulating cell proliferation, apoptosis, and migration (30). Studies have shown that sphingosine-1-phosphate (S1P) promotes the invasion and metastasis of breast cancer cells by activating its receptors (31). Furthermore, sphingolipid metabolism is closely linked to immune regulation in the tumor microenvironment. For example, overexpression of sphingosine kinase 1 (SPHK1) can suppress the antitumor activity of immune cells (32). The abnormal sphingolipid metabolism observed in this study may indicate the activation of sphingolipid signaling pathways in breast cancer patients, providing potential grounds for targeted therapeutic strategies aimed at sphingolipid metabolism. The disruption of propionate metabolism may reflect an imbalance in short-chain fatty acid metabolism in breast cancer patients. Propionate, a key intermediate in energy metabolism, can be converted into propionyl-CoA and further enter the tricarboxylic acid (TCA) cycle to provide energy for the cells (33). However, in breast cancer, mitochondrial function is often impaired, leading to a reprogramming of energy metabolism pathways (34). Abnormal propionate metabolism may weaken the energy supply to breast cancer cells and impair their metabolic adaptation, thereby promoting tumor progression. Additionally, short-chain fatty acids (such as propionate) have been shown to play a crucial role in immune regulation, and their metabolic imbalance may affect immune responses in the tumor microenvironment, thereby influencing the initiation and progression of breast cancer (35).

Notably, this study utilized saliva as a sample for early non-invasive breast cancer diagnosis. Compared to traditional methods like blood and tissue biopsies, saliva collection is more convenient, non-invasive, and better suited for large-scale screening. Additionally, saliva collection does not require specialized medical personnel, reducing patient compliance issues and healthcare costs, and improving the efficiency of early breast cancer screening. The results further confirm the potential of saliva metabolomics in breast cancer diagnosis. By conducting LC-MS/MS untargeted metabolomics analysis of saliva from breast cancer patients and healthy controls, we successfully identified a series of key metabolites closely associated with breast cancer, supporting saliva as a potential carrier for early detection biomarkers. Furthermore, it provides important scientific evidence for future non-invasive cancer screening. Future studies could combine multi-omics analysis (e.g., proteomics, transcriptomics) to explore the saliva metabolomic features of breast cancer subtypes, enhancing diagnostic accuracy and clinical translation value.

This study is subject to certain limitations. Firstly, the relatively small sample size may limit the generalizability of the findings. Consequently, future research is planned to include larger-scale, multicenter cohort studies to validate these results, ensuring their robustness and broader applicability. Secondly, although various statistical methods were employed for metabolite screening and pathway analysis, cell or animal experiments were not conducted to further validate the functions of these metabolites. Future studies could combine molecular biology experiments to further explore the mechanisms through which key metabolites influence the development of breast cancer. Additionally, this study did not differentiate between breast cancer subtypes, and metabolic characteristics may vary significantly across subtypes. Therefore, future research should explore the application value of saliva metabolomics in different breast cancer subtypes. In conclusion, this study demonstrates that LC-MS/MS-based untargeted saliva metabolomics analysis effectively identifies breast cancer-related metabolic features and selects a series of potential biomarkers. KEGG pathway analysis revealed the potential roles of metabolic pathways such as fatty acid biosynthesis, caffeine metabolism, and choline metabolism in breast cancer, providing new research directions for exploring the metabolic mechanisms of breast cancer. Furthermore, this study is the first to validate the feasibility of saliva metabolomics in non-invasive breast cancer screening, offering a new approach for future clinical testing. Future studies could expand the sample size, integrate molecular biology experiments, and explore the metabolic characteristics of different breast cancer subtypes to further advance early diagnosis and the optimization of personalized treatment strategies.

## Data Availability

The original contributions presented in the study are included in the article/**Supplementary Material**. Further inquiries can be directed to the corresponding author.

## Supplementary material

Supplementary Figure 1 PCA score plot of the QC samples.

Supplementary Table 1 Clinic characteristics of the validation set. p-value derived from t-test for continuous variables and Chi-square test for categorical variables comparing BC patients vs. Healthy Controls. N/A: Not Applicable.
