# Supplementary material for: Identification of potential biomarkers for breast cancer based on salivary metabolomics
Source: Front Oncol. 2025 Oct 10;15:1655213. doi: 10.3389/fonc.2025.1655213 (PMC12549245; doi:10.3389/fonc.2025.1655213)
Supplement: Supplementary Figure 1 — PCA score plot of the QC samples. [file DataSheet1.docx]

**Supplementary materials**

Table S1: Clinic characteristics of the validation set

| **Characteristic** | **BC Patients (n=52)** | **Controls (n = 52)** | **p-value** |
| --- | --- | --- | --- |
| Ages | 42.3 ± 9.7 | 41.1 ± 8.5 | 0.61 |
| BMI (kg/m²) | 28.6 ± 3.2 | 28.0 ± 3.0 | 0.44 |
| Ethnicity | Chinese | Chinese |  |
| Gender (Male/Female) | 0/52 | 0/52 |  |
| Menopausal Status, n (%) |  |  | 0.40 |
| - Premenopausal | 6 (11.5%) | 9 (15.4%) |  |
| - Postmenopausal | 46(88.5%) | 43 (84.6%) |  |
| Clinical stage |  |  | N/A |
| Early stage (I-II) | 50 (I:15, II:35) | N/A |  |
| Advanced stage (III-IV) | 2 (III:2, IV:0) | N/A |  |
| TNM status |  |  | N/A |
| Tumor status (T) | T1:23, T2:27, T3:0, T4:2 | N/A |  |
| Regional lymph node status (N) | N0:29, N1:10, N2:10, N3:3 | N/A |  |
| Distant metastasis status (M) | M0:50, M1:2 | N/A |  |

*p-value derived from t-test for continuous variables and Chi-square test for categorical variables comparing BC patients vs. Healthy Controls. N/A: Not Applicable.*


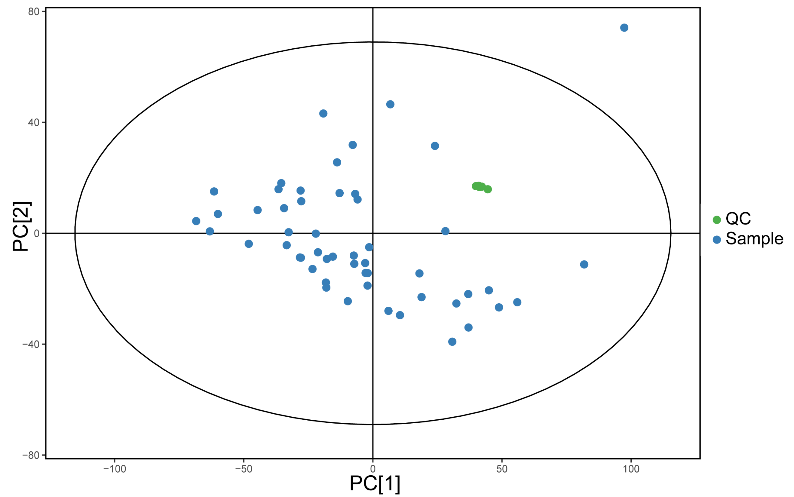


**Figure S1. PCA score plot of the QC samples.**
